# Intravesical Contrast-Enhanced MRI: A Potential Tool for Bladder Cancer Surveillance and Staging

**DOI:** 10.3390/curroncol30050350

**Published:** 2023-04-30

**Authors:** Pradeep Tyagi, Chan-Hong Moon, Marc Connell, Anirban Ganguly, Kang Jun Cho, Tatum Tarin, Rajiv Dhir, Biatta Sholosh, Jodi Maranchie

**Affiliations:** Department of Urology, School of Medicine, University of Pittsburgh, Pittsburgh, PA 15213, USA

**Keywords:** bladder cancer, staging, MRI, intravesical, gadolinium

## Abstract

This review article gives an overview of the current state of the art of bladder cancer imaging and then discusses in depth the scientific and technical merit of a novel imaging approach, tracing its evolution from murine cancer models to cancer patients. While the poor resolution of soft tissue obtained by widely available imaging options such as abdominal sonography and radiation-based CT leaves them only suitable for measuring the gross tumor volume and bladder wall thickening, dynamic contrast-enhanced magnetic resolution imaging (DCE MRI) is demonstrably superior in resolving muscle invasion. However, major barriers still exist in its adoption. Instead of injection for DCE-MRI, intravesical contrast-enhanced MRI (ICE-MRI) instills Gadolinium chelate (Gadobutrol) together with trace amounts of superparamagnetic agents for measurement of tumor volume, depth, and aggressiveness. ICE-MRI leverages leaky tight junctions to accelerate passive paracellular diffusion of Gadobutrol (604.71 Daltons) by treading the paracellular ingress pathway of fluorescein sodium and of mitomycin (<400 Daltons) into bladder tumor. The soaring cost of diagnosis and care of bladder cancer could be mitigated by reducing the use of expensive operating room resources with a potential non-surgical imaging option for cancer surveillance, thereby reducing over-diagnosis and over-treatment and increasing organ preservation.

## 1. Introduction

Bladder cancer (BCa) is one of the five most common cancers worldwide and is the most expensive cancer to treat, at USD 65,000 to 180,000 per patient, with an annual cost burden of USD 4 billion in the USA [1,2]. Given that the average age of diagnosis is 74 years, the gradual aging of populations in economically developed countries is also raising the prevalence of bladder cancer [3]. In the USA alone, new BCa cases are estimated to be 81,180, with estimated deaths at 17,100 for the year 2022 [3]. Bladder cancer (BCa) is the second most common neoplasm of the urinary tract, and based on data from the National Cancer Institute [3], an estimated 712,644 people were living with BCa in the United States as of 2019. Using those pre-pandemic numbers as a baseline, and after accounting for an average annual addition of ~81,000 new cases [3], we estimate that the number of Americans living with BCa in the country could cross the grim milestone of one million in 2023.

**Figure 1:**
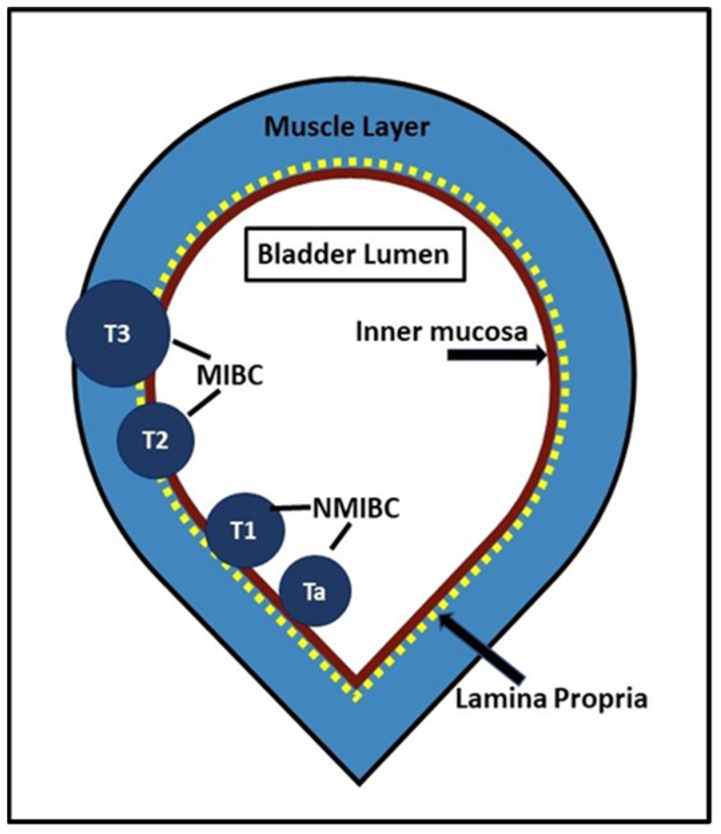
Urothelial carcinoma is staged by its depth of penetration into the three layers of the bladder wall (the inner mucosa, lamina propria and outer muscularis propria). Four bladder tumor stages are recognized: superficial neoplasm, also called non-muscle-invasive bladder cancer (NMIBC- Tis, Ta, and T1), muscle-invasive bladder cancer (MIBC), involving partial wall infiltrating neoplasms (T2), total wall infiltrating neoplasms [T3a (perivesical invasion on histology), T3b (perivesical invasion large enough to be seen on imaging)], and neoplasms involving other pelvic organs (T4).

The cost of the diagnosis and care of bladder cancer is soaring due to several factors: the rise in prevalence, a high recurrence rate of 75–80% [4], and the dependence of the standard of care on expensive operating room resources for BCa staging via transurethral resection of bladder tumor (TURBT). Over 98% of BCas are histologically confirmed to be urothelial cell carcinomas. Urothelial cell carcinomas are histologically stratified into cancers of low and high grade, and further sub-divided into non-muscle-invasive (NMIBC) and muscle-invasive (MIBC) categories depending on the penetration of the urothelial cancer cell into the different layers of the bladder wall (Figure 1). Stage distinction is a critical factor in clinical decision-making, as the treatment goal of low-grade NMIBC with indolent disease history is to reduce the local recurrence and to arrest progression to muscle-invasive disease. Around a third of NMIBCs present with high-grade lesions, and 20–25% of these can progress to muscle invasion (MIBC) within 5 years of TURBT [5]. MIBC is treated with neoadjuvant chemotherapy followed by complete removal of the bladder, whereas more superficial tumors (NIMBC) are amenable to bladder-sparing therapy.

Since a large majority of older adults [3] are only afflicted with superficial, exophytic tumors, with 5-year survival of 96%, an imaging technique capable of detecting the onset of muscle invasion may be a non-surgical staging option for virtual surveillance of indolent disease for preserving organ function and informing decision-making. Imaging can reduce the cost of surveillance, provide objective measures for early prediction of BCa aggressiveness and recurrence, to prevent delays in identifying MIBC patients for aggressive treatment of cystectomy-curative therapy and MIBC patients who may benefit from neoadjuvant chemotherapy [5]. Thus, an imaging technique with superior soft-tissue resolution may minimize the risk of under-treatment or over-treatment, saving lives and healthcare costs. This review gives an overview of the current state of the art of bladder cancer imaging, and then discusses in depth the scientific and technical merit of a novel imaging approach, by tracing its evolution from murine cancer models to cancer patients.

### Cost of Care

There are a million patients currently living with BCa in the USA, and each one of them will require a costly regimen of routine cystoscopy and a lifetime of continuous surveillance by cystoscopy in order to counter the high BCa recurrence rate of 80% after TURBT. BCa staging is the costliest component of BCa care, and staging determines the need for radiation and chemotherapy to prevent recurrence. While radical cystectomy remains the gold standard for staging accuracy, the accuracy of pathologic staging by TURBT is dependent upon complete resection of the tumor, and a high error rate of 25% [6] necessitates repeat TURBT for any high-grade NMIBC tumor found to be pathologic stage T1, in order to confirm the absence of muscle invasion prior to bladder sparing therapy [7]. Furthermore, distant metastases can develop in 12% [8,9,10] of patients, depending on whether the tumor has an intermediate or high risk of progression. Combined for all stages, the 5-year relative survival rate is 77%, which declines further with the increased passage of time after diagnosis. Furthermore, delays in elective procedures such as TURBT enforced by full utilization of operating room resources during pandemic highlight the unmet need of innovation for BCa staging.

## 2. Current Challenges

While cystoscopy is efficient in detecting exophytic tumors, it has a limited ability to image the subsurface tissue, which hinders cystoscopic discrimination of bladder tumors from a wide variety of non-neoplastic conditions [11] that can also thicken the bladder wall. BCas are frequently multifocal, and the origin and multifocality for recurrence can be traced to around 200–300 clonal stem cells [12] in the basal cell layer of uroepithelium that give rise to the transitional cell layer, or are associated with acute edema or hyperemia, which can mask the extent of disease [13]. Even with a solitary tumor, MIBC has been found to be under-staged by TURBT in up to 50% of cases, according to some studies [14].

### 2.1. Imaging Modalities

**Figure 2:**
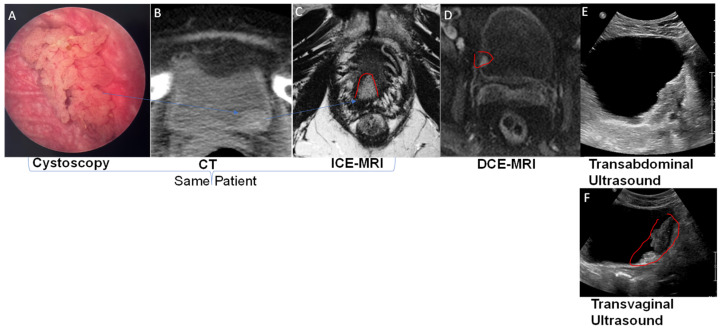
(**A**–**C**): A cystoscopically confirmed superficial tumor was imaged by CT and then by intravesical contrast-enhanced (ICE)-MRI after the instillation of the Gadolinium-based contrast agent GBCA and (Ferumoxytol) for positive and negative contrast, respectively, creating a clear demarcation of the bladder tumor (red circle) shape, size, and depth of penetration. (**D**–**F**): Dynamic contrast-enhanced magnetic resonance imaging (DCE-MRI) after intravenous injection of GBCA could image the NMIBC prior to TURBT, whereas the poor soft tissue resolution of CT is only good for estimating gross tumor volume, and the image resolution of transvaginal ultrasound (**F**) is superior to transabdominal ultrasound (**E**).

Abdominal sonography (Figure 2E,F) [15] and radiation-based imaging modalities such as computed tomography (CT) (Figure 2B) can measure bladder wall thickening secondary to idiopathic, infectious, or non-infectious inflammatory conditions [16], but their poor tissue resolution presents a challenge. Abdominal sonography (Figure 2E) [17,18] has been shown to increase the risk of over-staging NMIBC and under-staging MIBC [19,20], though transvaginal sonography in female bladder cancer patients (Figure 2F) achieves a higher spatial resolution by reducing the physical distance between the ultrasound probe and the diseased area on the bladder wall. Improvement in real-time spatial resolution is the motivation for the development of micro-ultrasound (MUS) technology [21]. A recent study reported that MUS [22] using a 29-MHz side-fire transducer accurately predicted muscle invasion in 5 out of 7 BCa patients, or accuracy of 71.4%, and differentiated NMIBC in 14 patients.

The need for a high radiation dose and an intravenous ionic contrast agent makes CT unsuitable for regular surveillance of BCa [19]. CT is only good for estimating the gross tumor volume, as its poor soft tissue resolution makes it incapable of differentiating between superficial BCa and muscle invasion. Since the available techniques suffer from a ~50% failure rate [23] in detecting deeper muscle invasion, imaging modalities are inadequate in both assessing tumor aggressiveness [24] and in detecting carcinomas in situ (CIS) [6], which adversely impacts [25] the early identification of BCa patients most likely to benefit from chemotherapy or immunotherapy [26]. The superior soft tissue resolution of magnetic resonance imaging (MRI) offers several advantages (Figure 2D), but challenges remain in its use for BCa staging.

### 2.2. Magnetic Resonance Imaging (MRI)

**Figure 3:**
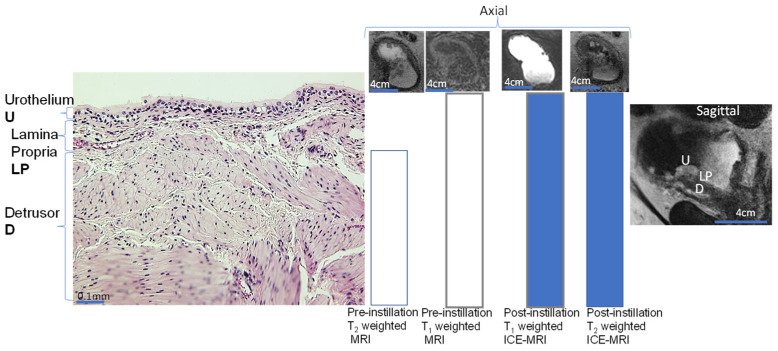
Schematic illustration of mammalian bladder wall anatomy pre and post instillation of contrast mixture for ICE-MRI. Only the muscle layer is visible in pre-contrast T2-weighted MRI (MRI adjacent to histology) compared to the whole bladder wall in T1-weighted MRI acquired in axial orientation. The cystoscopically confirmed muscle-invasive multifocal tumor is more clearly visible in ICE-MRI with all three layers visible in T2-weighted 2D-TSE (TR/TE 4000/100 ms, with a bigger voxel volume of 0.625 × 0.625 × 3 mm^3^ vs. 0.67 × 0.67 × 1 mm^3^, and twice the number of signal averages (NSA) than in T1-weighted 3D FLASH (TR/TE 5.24/1.86 ms) at a flip angle (FA) of 20°. Bladder wall segmentation into the urothelium (U), lamina propria (L) and detrusor D is more clearly visible in the magnified view on sagittal orientation with ICE-MRI. Scale bar is there for comparison with axial view.

MRI affords good soft tissue contrast in multiplanar images of visceral organs without the use of ionizing radiation [27], but conventional MRI without contrast enhancement has notable drawbacks [28], as illustrated by unenhanced conventional MRI in Figure 3.

**Figure 4:**
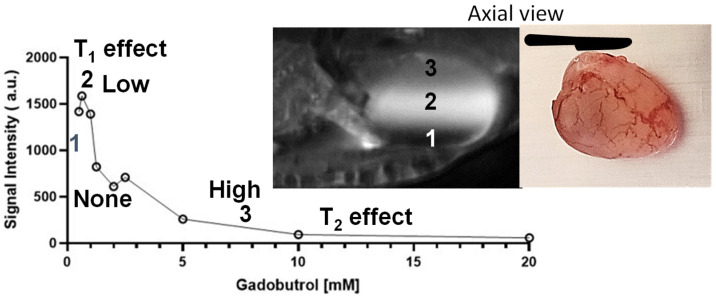
Pseudolayering observed in a mouse bladder lumen during multi-slice T1-weighted DCE-MRI, after intravenous injection of Gadobutrol (0.1 mm/kg) manifests the concentration-dependent T1- and T2-shortening effect of Gadobutrol on measured signal intensity (arbitrary units, a.u), with T1 relaxivity dominating at lower concentrations (<1 mM), and T2 relaxivity dominating at higher concentrations (>5 mM).

The MR contrast of cancer foci with respect to normal areas (Figure 2, Figure 3 and Figure 4) depends upon the differences in proton-spin density, magnetic susceptibility, water proton T1 (spin–lattice relaxation time), T2 (spin–spin relaxation time), magnetic susceptibility, molecular diffusion, and perfusion. In unenhanced conventional MRI performed without any injection or instillation of Gadolinium based contrast agent (GBCA), bladder tumor appears hypointense to urine and isointense or slightly hyperintense compared to the muscle layer in T2-weighted turbo spin echo (TSE) images (Figure 3). The relatively long intrinsic relaxation time (T1) of both urine and the bladder wall [29] leads to poor visualization of the rodent bladder wall [30,31] and the human bladder wall [32] in T1-weighted FLASH at an FA of 20° (Figure 3), prior to injection or instillation of GBCA.

**Figure 5:**
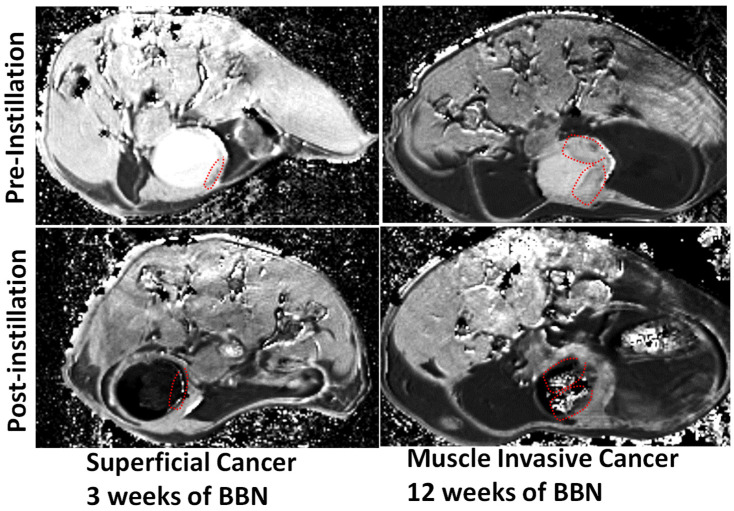
Proof of the principle for bladder cancer surveillance and staging by multi-slice, T1-weighted ICE-MRI in axial orientation of 6-week-old B6D2F1 female mice fed carcinogen, N-butyl-N-4-hydroxybutyl nitrosamine (BBN), ad libitum, in drinking water (0.05% *w/v*) for up to 12 weeks. Tumoritropic infiltration of Gadobutrol accomplishes non-surgical surveillance of small polyps (red dotted circle) at 3 weeks, and stages muscle-invasive cancer (red dotted circle) by 12 weeks without transurethral resection by acquiring ICE-MRI under isoflurane anesthesia. Transurethral instillation of 0.05 mL Gadobutrol (4 mM) and Ferumoxytol (5 mM) for a 30 min period accomplishes voxel-wise T1 mapping at a variable TR of 400–7500 ms, a TE of 6.5 ms, a slice thickness 0.8 mm, an FOV of 28 × 28 mm^2^, an acquisition matrix 218 × 218, and an NSA = 1.

An MRI image (Figure 2, Figure 3, Figure 4 and Figure 5) is made up of many pixels (two-dimensional (2D) units of image space) corresponding to a three-dimensional (3D) volume called a voxel, defined by a field-of-view (FOV) of 160 × 160 mm^2^, an acquisition matrix of 256 × 256 and a slice thickness of 3 mm for the multi-slice T2-weighted turbo spin echo image (the leftmost MRI image of Figure 3), with fat suppression in a voxel volume 0.625 × 0.625 × 3 mm^3^, a 3 mm interslice gap, a repetition time (TR) of 4000 milliseconds (ms), an echo time (TE) of 100 ms, and a number of signal averages (NSA) = 2 for 30 slices acquired in 92 s [11]. An NSA = 1 and a smaller voxel volume of 0.67 × 0.67 × 1 mm^3^ was used to acquire 72 contiguous slices of 1 mm slice thickness in 23.30 s at each flip angle (FA) from 3° to 22°, using a fast low-angle shot 3D volume-interpolated breath-hold examination (VIBE) sequence for T1-weighted MRI; this can be seen in Figure 3, with a TR/TE of 5.24/1.86 ms and an FOV 154 × 259 mm^2^, and rectangular matrix of 192 × 80. Accordingly, the post-instillation T2-weighted MRI shows a clearer visualization of the tumor on the lateral bladder wall, with maximum spatial resolution and higher signal-to-noise ratio than T1-weighted MRI (Figure 3). Importantly, the hypointense signal of the lateral tumor on T2-weighted axial scans (Figure 3) and slight hyperintensity on T1-weighted FLASH images, acquired without the presence of GBCA in pre-instillation intravesical contrast-enhanced MRI (ICE-MRI) (Figure 3), agrees with the consensus view of the vesical imaging–reporting and data system, (VI-RADS) [9] on the shortcomings of unenhanced MRI. The bright signal of urine obscures the signal from the urothelium and lamina propria to engender an inaccurate measurement of bladder thickness and tumor dimensions in T2-weighted MRI compared to T1-weighted MRI (Figure 3).

### 2.3. DCE-MRI

DCE-MRI following intravenous injection of GBCA visualizes the bladder wall (Figure 2D) in three layers: an inner thin layer of low intensity (mucosa), a middle layer of marked enhancement (lamina propria LP), and a thick outer layer of intermediate intensity (muscularis propria smooth muscle) akin to the segmentation shown in sagittal orientation for ICE-MRI. Upon coming into contact with the paramagnetic, GBCA [33], the T1 water relaxation rate (R1 = 1/T1 relaxation time measured in milliseconds (ms) of a voxel in the tumor increases in linear proportion to the tissue concentration of GBCA in that voxel [34,35]. Tumor-associated angiogenesis delivers a higher concentration of injected GBCA to voxels containing tumor(Figure 2D) compared to normal bladder wall, on the basis of a linear equation [31]: ΔR1 = R1 − R10 = r1[Gd], where R10 and R1 are the pre-contrast (baseline) and post-contrast T1 water relaxation rates (1/T1) of a voxel [34,35], respectively, and r1 is the relaxivity [30] of the T1 relaxation rate constant for GBCA.

As a result, the contrast enhancement in DCE-MRI (middle panel Figure 2) is a function of GBCA concentration delivered to the tumor via arterial perfusion [34,35], but the rapid washout leads to transient enhancement of the tumor (<3 min) [36]. Akin to DCE-MRI images of Figure 2D), post-instillation FLASH images acquired by ICE-MRI at the same FA of 20° could also clearly visualize the tumor (Figure 2C and Figure 3). The imaging parameters remained the same from pre-instillation to post-instillation of the contrast mixture. Contrary to transient (<3 min) enhancement of tumor by DCE-MRI [9,34,35], ICE-MRI enables clear visualization of high-grade urothelial carcinomas (Figure 2 and Figure 5), with a high signal-to-noise ratio for high resolution imaging, without entailing the risk of allergy and heavy metal toxicity with repeated DCE-MRI [37], because there is no injection of intravenous contrast [11]. The in-plane resolution of 0.67 mm at 3T with fat and water suppression (TR/TE of 5.24/1.86 ms) in ICE-MRI (Figure 2 and Figure 3) improves the spatial resolution of 0.9 mm achieved in past studies [38]. Fast 3D acquisition methods such as the volume-interpolated breath-hold examination (VIBE) sequence significantly reduce motion artifacts without requiring patients to hold their breath.

Several studies on BCa have found that DCE-MRI (Figure 2D) is dramatically superior to CT (Figure 2B) by 30–40% in preoperative primary tumor (T stage) staging of BCa [39,40,41], and achieves 87–92% accuracy in the detection of MIBC, with a false positive rate of only 8.3% [39,40,41]. Despite a superior performance to CT, DCE-MRI is still not the standard of care for BCa staging, because GBCA injection entails the risk of allergic reaction and of heavy metal toxicity upon repeated use [37,42,43], which would be required for BCa surveillance. Intravenous injection of GBCA also raises healthcare costs, and GBCA injection is contraindicated in patients with renal insufficiency, if their glomerular filtration rate is <30, a state commonly seen in BCa due to urinary tract obstruction by the tumor.

GBCA injected for DCE-MRI is also excreted unchanged in urine, and its continuous accumulation in the lumen [44,45,46] rapidly reduces the image contrast between the lumen and bladder wall (Figure 4) [32,47,48] due to pseudolayering, which leaves a rapidly closing window for bladder tumor imaging by DCE-MRI (<3 min) [34,35,36]. While the relationship between GBCA concentration and T1 relaxation rate is linear [30,31], a non-linear relationship between GBCA concentration and the signal intensity (Figure 4) [45,49,50] results in the phenomenon of pseudolayering [45,51,52] (inset of Figure 4) due to the concentration-dependent T1- and T2-shortening effect of Gadobutrol [45]. Pseudolayering was noted on the DCE-MRI of a male mouse anaesthetized under isoflurane at 7T, with a TR/TE = 200/5.23 ms, 9 slices, an acquisition matrix of 128 × 128 and a field-of-view of 20 × 20 mm^2^, a slice thickness 0.8 mm, an NSA = 1, a temporal resolution of 12 s and an in-plane resolution of 156 μm, more than 4 times lower than in-plane resolution of 670 μm at 3T.

## 3. Intravesical Contrast-Enhanced MRI (ICE-MRI)

**Figure 6:**
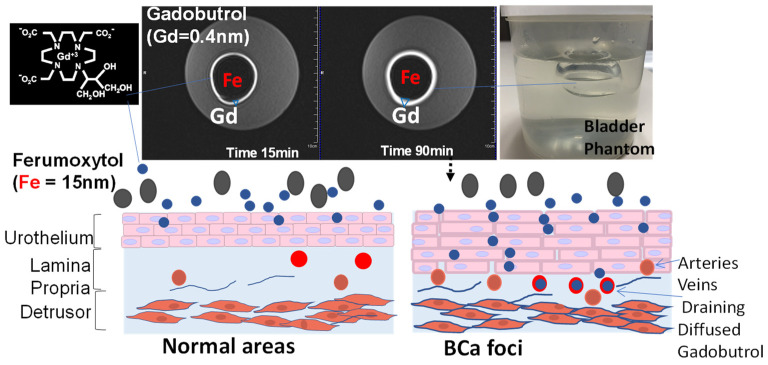
MRI of a bladder phantom. The principle of Stokesian diffusion dictates passive, isotropic, time-dependent diffusion of Gadobutrol (0.4 nm) into >20 times bigger pores of polyacrylamide gel over 90 min (indicated by bigger blue∇ in the right image). The bright ring increasing from 15 to 90 min, which depicts paracellular Gd diffusion, is thrown into sharp relief by the dark cavity mimicking the luminal retention of a larger-sized Ferumoxytol (Fe 15 nm), with its larger magnetic moment decaying the Gadobutrol signal within the lumen.

Given that contrast enhancement of cancer foci in DCE-MRI is a function of GBCA concentration delivered by perfusion, we wondered if GBCA delivered by urothelial diffusion to tumor overcomes the above-stated drawbacks of DCE-MRI. This question inspired an intravesical offshoot of DCE-MRI with minimal invasiveness, called ICE-MRI [30,31,53,54]. Since past studies have reported that instillation of GBCA does not offer any advantage in bladder cancer staging over DCE-MRI [55], ICE-MRI is centered on bladder instillation via a transurethral catheter of two FDA-approved agents, Gadobutrol and Ferumoxytol, as a mixture [31,53,54]. Instead of arriving to the tumor by perfusion, ICE-MRI is predicated on the diffusion of instilled Gadobutrol along the downhill concentration gradient from the lumen to reach the highest concentration in the inner layer of the mucosa [56] for positive contrast and negative contrast in the lumen, resulting from the luminal retention of Ferumoxytol (Figure 5 and Figure 6). ICE-MRI ensures uniformity of contrast in lumen by picking a Gadobutrol concentration from Figure 4, which is insensitive to any dilution from fresh urine. Simply stated, the ICE-MRI seeks to repurpose FDA-approved agents for differential contrast enhancement of neoplastic and non-neoplastic lesions on the bladder wall by leveraging the published histological differences between neoplastic [57,58] and non-neoplastic lesions [59], and the structural differences in urothelial permeability [60]. In addition, since the cellularity of the tumor reduces the extracellular space available for Gadobutrol diffusion relative to inflamed areas [11], there is bound to be differences in contrast enhancement.

Figure 2 and Figure 5 illustrate that ICE-MRI [53,54] provides stable positive contrast in rodent and human bladders, and the period of artifact-free visualization can be extended nearly 10-fold compared to DCE-MRI [36]. On the other hand, the reduced bioavailability of Gadobutrol dose 1 mmol (seven times lower than the recommended intravenous dose) [27,53,55] instilled into bladder eliminates the inherent risks of heavy metal toxicity and allergic reaction associated with GBCA injection [61,62]. Preclinical findings of a dark lumen adjacent to a bright bladder wall [30,31,54], generated by ICE-MRI at 7T [54,63], and a 9.4T animal scanner [30] were reproduced in the T2-weighted turbo spin echo images acquired at clinical scanner 3T (Figure 2C and Figure 3).

The graded decline in the signal intensity across bladder wall tissue layers (Figure 3 and Figure 5) manifests the logarithmic decline of diffusing Gadobutrol concentration [31] from the mucosa to deeper tissue layers. The logarithmic decline in diffused Gadobutrol concentration [31] stems from homeostatic venous clearance of any instilled drug reaching mucosa, [64]. Angiogenesis of the bladder tumor [63] augments the venous drainage of diffused Gadobutrol to accentuate the concentration gradient which may accelerate the Fickian diffusion of instilled Gadobutrol. As a result, the intensity of enhancement descends from aggressive cancer lesion > indolent cancer lesion > non-cancerous bladder wall (Figure 5). Cancerous lesions [65,66] on the luminal surface of the bladder are characterized by a disrupted tight junction barrier [67], and tumoritropic infiltration [63] of GBCA generates localized enhancement (Figure 2C). An instilled volume of 0.05 mL of contrast mixture in the ICE-MRI of the murine bladder was based on the volume instilled in past mouse studies [31].

### 3.1. Past Attempts of Adding Negative Contrast to Bladder

Since pseudolayering in the bladder lumen with DCE-MRI results from a lack of uniform contrast in the lumen, several group resorted to insufflation air [68] or instillation of Ferumoxytol [69] to ensure uniformity of contrast in the human bladder lumen during DCE-MRI. While Ferumoxytol instillation alone was directly tried in humans, air insufflation was also tried in a mouse bladder together with GBCA instillation [70]. However, both approaches failed to offer any advantage in improving the accuracy of BCa staging. As extensively reported by several groups [55,71], bladder instillation of just GBCA alone without the inclusion of a negative MR contrast (i.e., a hypo-intensity signal) is unable to achieve a clear visualization of bladder wall, which is a prerequisite for BCa staging. Learning from the lessons of past studies, we proposed the replacement of the intravenous injection with the instillation of positive (GBCA) alongside negative contrast agents, in order to remove the barriers to the adoption of MRI for BCa staging and prognosis [36].

### 3.2. Principle

The innovation of ICE-MRI capitalizes on the inverse relationship between the diffusion rate and the Stokes–Einstein radius of instilled drugs. Stokesian diffusion dictates the paracellular diffusion of smaller Gadobutrol (with a Stokes–Einstein radius of 0.4 nm and a molecular weight of 604.7 Daltons), whereas the diffusion of larger-sized Ferumoxytol (with a Stokes–Einstein radius of 15 nm and a molecular weight of 731 kiloDaltons) is retarded. Gadobutrol [72] and Ferumoxytol [73,74,75,76] also differ in their magnetic moment for tumorotropic infiltration of Gadobutrol [70,77] to cause tumor enhancement [30,31], while luminal retention of Ferumoxytol darkens the bladder lumen [63] (Figure 5 and Figure 6), as the large magnetic moment of iron [73,74,75,76] induces local magnetic field inhomogeneities to dephase proton spins, causing signal decay within the bladder lumen (Figure 6) [53,54,78]. The restricted paracellular diffusion (Figure 6) of Ferumoxytol is analogous to the limited bladder adherence of instilled antibodies (~120 kiloDaltons) in the human bladder [79,80,81,82] and of inulin (5000 Daltons) in rat bladders [83]. Since Gadobutrol shortens the T1 relaxation time of either medium [30], pig bladder [84] or human bladder [53], in a concentration-dependent manner, ICE-MRI capitalizes on the linear relationship between Gadobutrol concentration and the concentration-dependent [30,31,54], differential changes in the T1 relaxation rate of cancerous foci and normal regions [30,31,53] to generate artifact-free, high-resolution 3D images suitable for “virtual histology” or non-surgical BCa staging for routine surveillance of tumor recurrence and progression [63].

### 3.3. Paracellular Path of Diffusion

ICE-MRI is predicated on the perturbed tight junctions [67,85] of bladder tumors relative to the normal areas that are shown to cause tumoritropic infiltration of small molecular weight drugs/dyes: such as mitomycin [64], fluorescein [86], methylene blue [87,88,89]. However, diffusion of high molecular weight radiolabeled probes [79,80,81,82] around tight junctions is slowed in accordance with the principle of Stokesian diffusion. Instilled GBCA is unlikely to enter umbrella cells as the transcellular permeability of umbrella cells is restricted, and GBCA is unable to enter even red blood cells upon injection [90]. The perturbed tight junctions [67,85] of cancer foci are known to compromise the urothelial barrier, which accentuates the passive diffusion of instilled GBCA in both rodent (Figure 5) [63,65,70,77] and human bladder (Figure 2 and Figure 3) [55], analogous to the diffusion of instilled polar dyes in preclinical [91,92,93] and clinical [86,87,88,89] studies. The differential signal enhancement of cancer foci by ICE-MRI replicates the results obtained with other radiation-free approaches [58,86]. Moreover, a higher ingress of instilled Gadobutrol into cancer foci (Figure 2, Figure 3, Figure 4 and Figure 5) corroborates earlier reports [34,35] and agrees with the higher serum uptake of instilled radiolabeled Na^+^ and urea [60] in bladder cancer patients, compared to urinary tract infection [60] and urinary retention patients.

### 3.4. Effect of Urinary Dilution on Image Contrast

Given that the physical gap of the apico-lateral tight junction [91,92,94] (Figure 2 and Figure 3) can be partly mimicked by the pore size of 12% polyacrylamide gel [95], we relied on that equivalence to study the paracellular diffusion [31] of Gadobutrol without the confounding influence (Figure 6) of bladder distension and bladder perfusion [84,96]. A spherical bladder-shaped cavity was molded with 12% polyacrylamide gel poured into a plastic container, which was wrapped by a 4-channel flexible receiver coil, for image acquisition using a multi-echo spoiled-gradient echo pulse sequence in 3T scanner (Siemens, BioGraph) Figure 6. We also visualized the concentration-dependent diffusion of Gadobutrol [96] using nine cylindrical cavities of gel phantom filled with ascending concentrations of Gadobutrol [0.5–20 mM] together with a fixed concentration of Ferumoxytol 0.1 mM. While the concentration gradient is critical for ensuring the paracellular diffusion [56,97] of instilled Gadobutrol, it also lowers the signal intensity in the mucosa more than in the lumen [71] and replicates the poor image contrast with the instillation of just Gadobutrol alone into mammalian bladder [54,55,70,71,77].

### 3.5. Clinical Translation of ICE-MRI from 7T to 3T

The ten-fold lower thickness of rodent bladder wall (~0.5 mm) compared to human bladder wall (~5 mm) requires a proportionally higher signal-to-noise ratio of ≥7T for imaging mouse bladder cancer (Figure 5) [63]. The clinical translation of ICE-MRI tackled differences in field strength and pulse sequences from spin echo at a higher field of 7T to gradient echo (FLASH) at 3T for human subjects (Figure 2 and Figure 3) by adjusting the concentration of instilled Gadobutrol and Ferumoxytol. Accordingly, to limit signal dephasing in bladder wall with the use of gradient echo for T1 weighted imaging, we lowered the Ferumoxytol concentration [84] from our past study [53] by raising the Gadobutrol concentration [84]. While a lower concentration of Ferumoxytol at 3T minimizes local magnetic field inhomogeneity, a higher concentration of Gadobutrol elevates the downhill concentration gradient [96] for faster Fickian diffusion and thereby facilitate rapid image acquisition of human subjects [11] up to ~15 min post instillation of the contrast mixture. Assuming a urine production rate of 1 mL/min during the post-instillation scanning period, the clinical data of Figure 2C reproduces the dark lumen predicted by Figure 4, as the Gadobutrol concentration of >5 mM exhibits a minimal change in signal intensity, even by resisting the dilution from urine excreted over the scanning period.

## 4. Clinical Protocol for ICE-MRI

The superior imaging characteristics of the rodent bladder imaged with the 7T [54] and 9.4T animal scanner [30] informed a small pilot clinical study of ICE-MRI on six human subjects to assess its safety [53] and feasibility at a clinical field strength of 3T. After acquiring the pre-instillation MRI with the standard pulse sequences [11] described for Figure 3, the subject laid in supine position on the scanner platform and was catheterized with an uncoated 14Fr urethral straight tip catheter to remove urine prior to 50 mL instillation of an extemporaneously prepared mixture of Gadobutrol (Gadovist, 1 mmol/mL) and Ferumoxytol (Feraheme 30 mg/mL), procured from Bayer Healthcare, Wayne, NJ and AMAG Pharmaceuticals Inc., Waltham, MA, respectively. Two agents were mixed in a 250 mL pouch of sterile water for irrigation, to reach the final concentrations of Gadobutrol and Ferumoxytol of 20 mM and 0.1 mM, respectively [53]. The shorter echo time <2 ms in the FLASH sequence minimizes the signal decay due to the T2* effects, and the exacerbation of B1 field inhomogeneity with gradient echo is minimized by instilling a lower concentration of Ferumoxytol. Instead of the recommended distension of the bladder by 300 mL for MRI [9], an instillation of only 50 mL for ICE-MRI was able to overcome the intrinsic drawbacks of T2-weighted MRl for bladder imaging [9], and the use of a lower instillation volume (≤50 mL) is also backed by intravesical pharmacokinetics [97] and preclinical studies [93,98].

After instillation, the subject was repositioned to the same position in the scanner as that used for the pre-instillation MRI [11] (Figure 3); the bladder was localized again for repeating the pre-instillation MRI protocol, involving T2-weighted turbo echo and T1-weighted 3D-FLASH VIBE with total scan time of 15 min pre-instillation and 15 min post-instillation of contrast mixture. The contrast mixture was well tolerated, and no adverse events were observed. Subjects took a ciprofloxacin dose by oral route at the end of the imaging as a prophylaxis against infection from the instillation procedure. The location and laterality of the lesion were recorded by a radiologist blinded to the disease history, cystoscopy, and pathology of the lesions. Lesions were categorized as mass or non-mass enhancement and correlated with histopathologic findings at TUR. Both regions of interest were manually segmented in three dimensions [11].

### Variable Flip Angle (VFA) for Mapping T1 Relaxation Time by ICE-MRI

T1 relaxation time is an intrinsic MR parameter which graphically represents the first order time constant required for longitudinal magnetization to reach ~63% of maximum signal intensity [99] after application of the radio pulse. The T1 relaxation time constant [100] of any tissue is an intrinsic physical parameter influenced by the structural and cellular organization of the bladder wall (Figure 5) [30,31].

We [53] and others [101] have used the spoiled gradient recalled echo VFA technique with 2D-FLASH in the past for rapid mapping of the T1 relaxation time of a human bladder mucosa in a single slice of 1544 ± 34.2 ms at 3T [53,54]. Since signal intensity (Figure 3) becomes a function of the tissue T1 at different FAs [102,103], the acquisition of a series of T1-weighted images at variable FAs allows the computation of the T1 relaxation time (T1). The value addition from multi-slice image acquisition for a T1-weighted 3-D VIBE sequence [34] over a single slice is best illustrated by the clear visibility and the boundary details of the tumor relative to normal areas (Figure 2 and Figure 3) at a constant TR of 5.2 ms and a TE of 1.8 ms. The clinical significance of volumetric T1-weighted imaging [34] over single-slice imaging is also highlighted by the separation of 10 slices, or ~10 mm, between the slices displaying the largest proportion of the BCa tumor [11].

ICE-MRI can sustain the enhancement of urothelial lesions with a graded decline in signal intensity for as long as the contrast mixture is held in the bladder [11]. ICE-MRI takes an average of the pre-instillation T1 relaxation rate for suspected lesions and normal regions of bladder and therefore, ICE-MRI is not dependent on the exact matching of the scans acquired before and after instillation of the contrast mixture. The average pre-instillation T1 of the bladder wall was used as a common parameter for computing the T1 relaxation rate of cancer foci and non-cancerous (normal) regions of the bladder wall [63]. The higher diffusion of instilled Gadobutrol into the lesioned area causes a dramatic change in the T1 relaxation rate of lesioned area [63] relative to non-lesioned area.

We did not image the bladder wall after withdrawing the contrast mixture to check how long it might take for the bladder wall to be cleared of the diffused Gadobutrol. Based on results obtained with the instillation of Gadobutrol (1 millimole), we envision that instillation of higher millimoles may transiently raise the residual tumor concentration of Gadobutrol, and may act as beacons for radiotherapy immediately after MRI instead of the endoscopic gold placement [104]. Since CT is poor in predicting the location of the tumor site [104], ICE-MRI could be potentially combined with radiation therapy for improved visualization of the tumor to reduce collateral radiation away from the cancer foci for preserving organ function in elderly.

## 5. Conclusions

An imaging technique capable of detecting the onset of muscle invasion could not only provide a lower-cost option for cancer surveillance in the elderly but could also meet the primary treatment goals of reducing disease progression, preserving organ function in patients deemed suitable for close surveillance, and avoiding overtreatment of indolent disease.

## Data Availability

Data are unavailable due to privacy or ethical restrictions.
